# Supplementary material for: Multilayer hazes over Saturn’s hexagon from Cassini ISS limb images
Source: Nat Commun. 2020 May 8;11:2281. doi: 10.1038/s41467-020-16110-1 (PMC7210256; doi:10.1038/s41467-020-16110-1)
Supplement: Supplementary file 1 — Supplementary Information [file 41467_2020_16110_MOESM1_ESM.pdf]

# Supplementary Information File

## Multilayer hazes over Saturn’s Hexagon from Cassini ISS limb images

By Sánchez-Lavega et al.

# Supplementary Figures

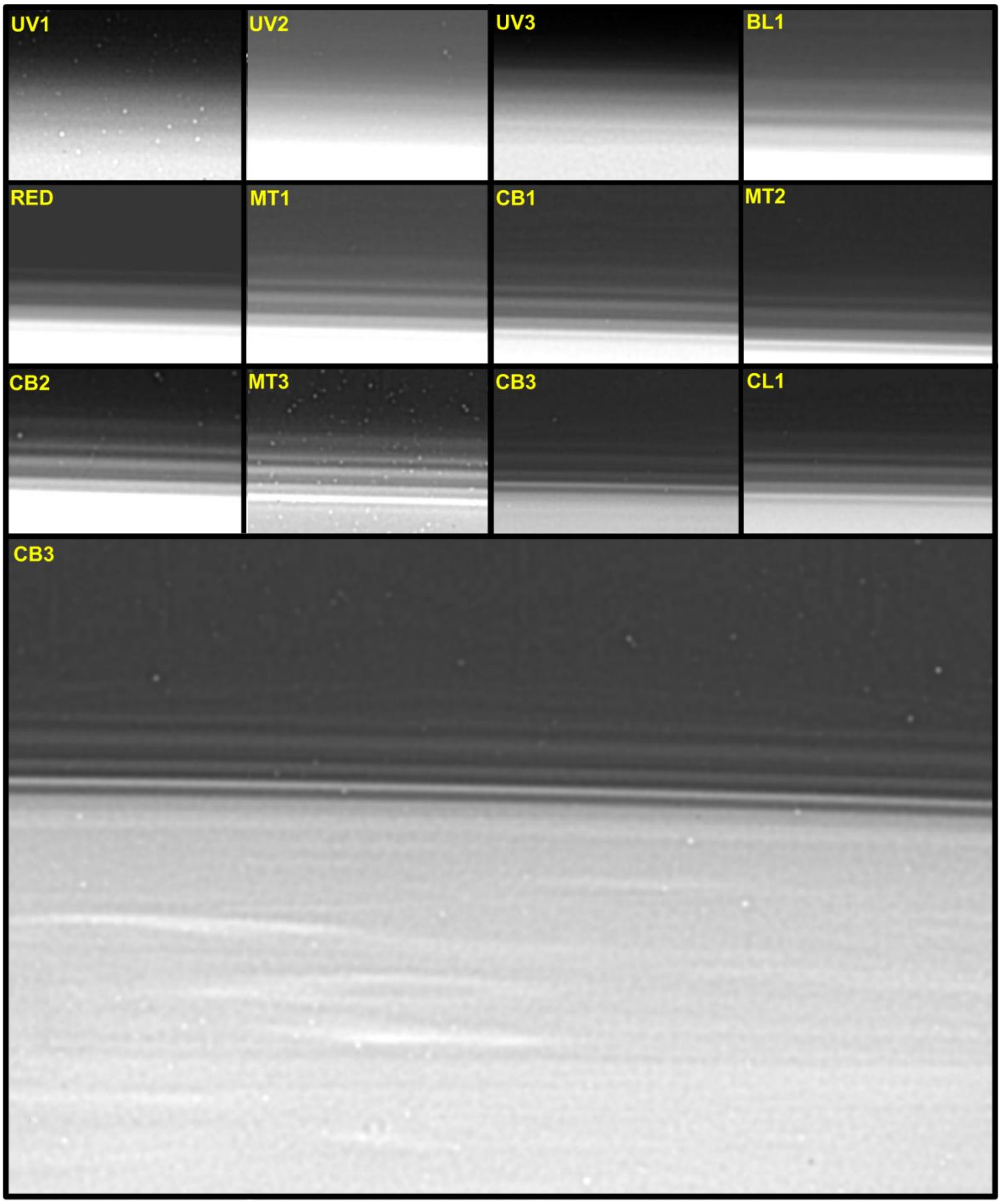


**Supplementary Figure 1. Cassini ISS images of the hexagon region hazes at different wavelengths.** Top: the identification follows the filter name and the data are given in Table S2. Bottom: Magnified image in CB3 to show the limb hazes and foreground isolated bright clouds overshooting the main haze layers.

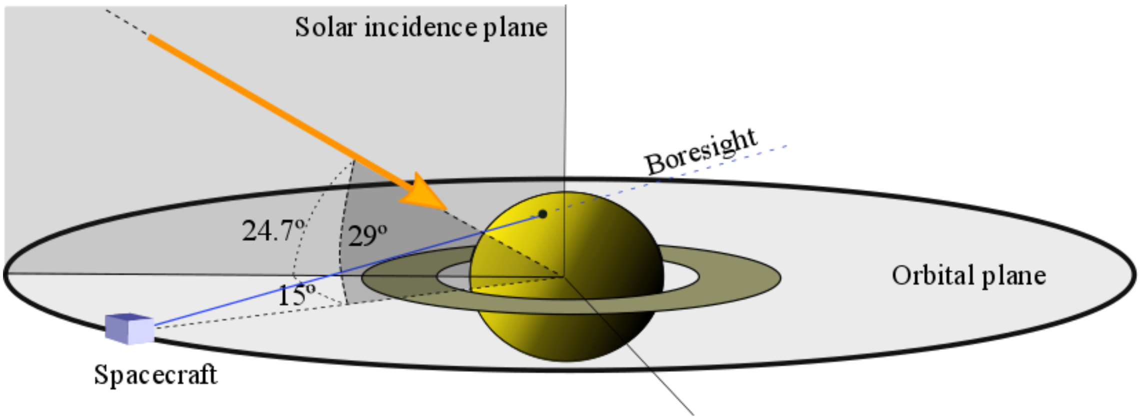


**Supplementary Figure 2. Geometry of the observation and angles.** Date of Cassini ISS images was June 15, 2015. The sub-spacecraft coordinates were: planetocentric latitude = 0.25° and System III Longitude = 285°. The sub-solar coordinates were: planetocentric latitude = 24.7° and System III Longitude = 300°. The phase angle was $\alpha$ = 29° (scattering angle $\theta$ = 151°). The spacecraft distance to the planet was 4.6 Saturn radius. The limb location (average of all images) was planetographic latitude 76.4° and System III longitude = 254°.

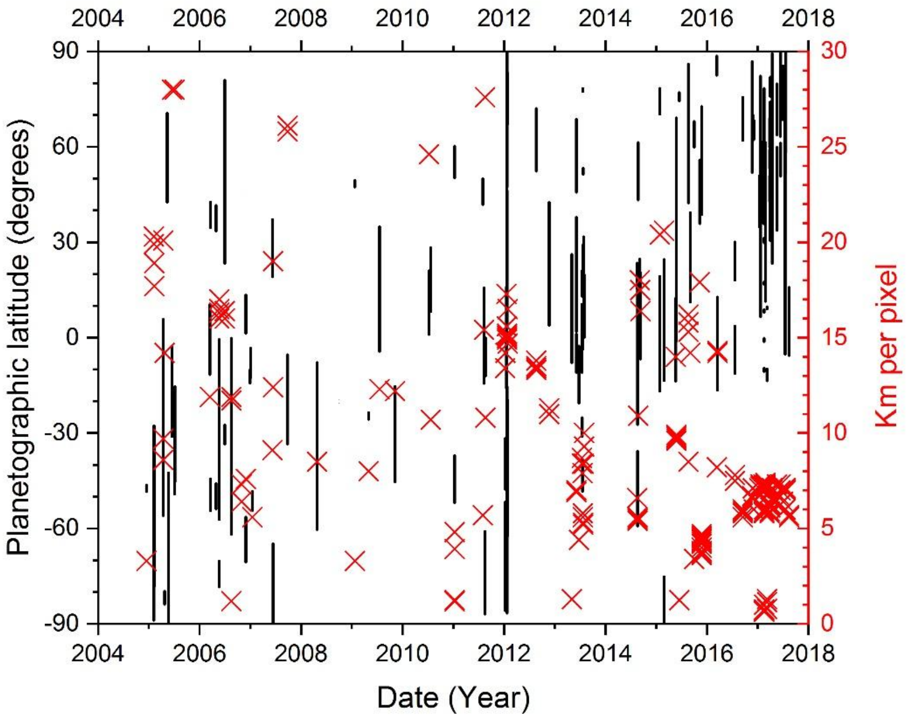


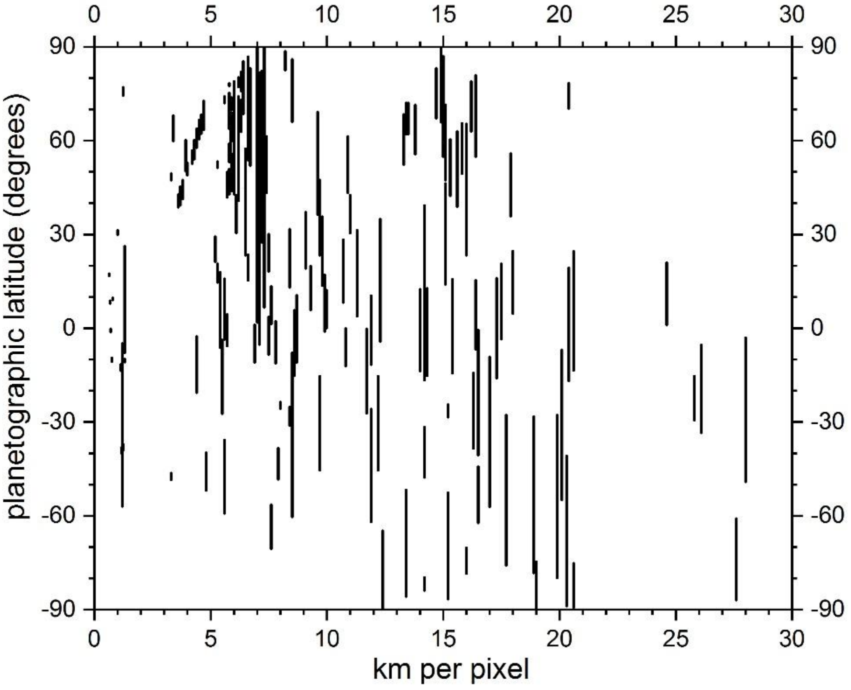


**Supplementary Figure 3. Cassini ISS NAC camera limb observations from December 2004 to September 2017.** Top: temporal sampling along this period of the latitude coverage (left axis) and the spatial resolution (right axis). Bottom: latitude coverage against spatial resolution (km/pixel).

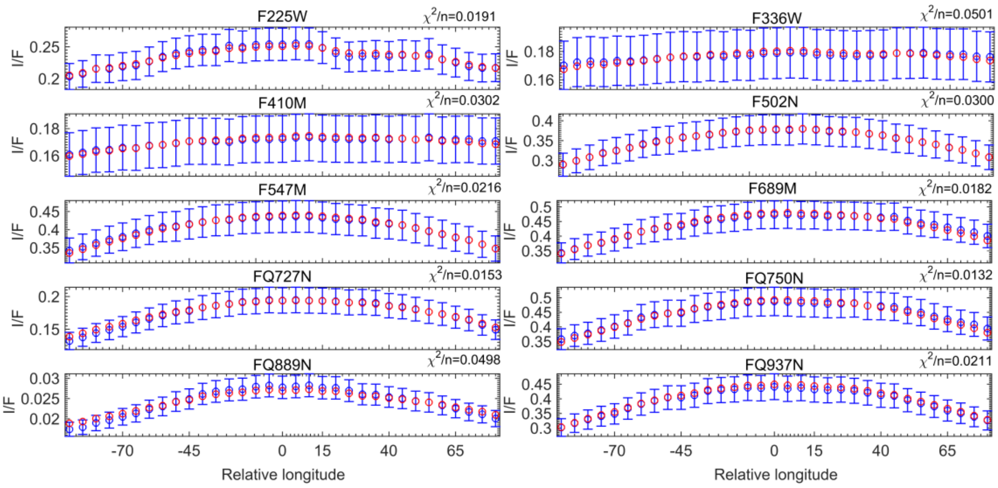


**Supplementary Figure 4. Reflectivity profiles.** Comparison of the center-to-limb reflectivity profiles at different wavelengths (filter indicated in the individual panels, see Table S1) from HST images from 29-30 June 2015 (blue circles with error bars) and from a radiative transfer model (red circles).

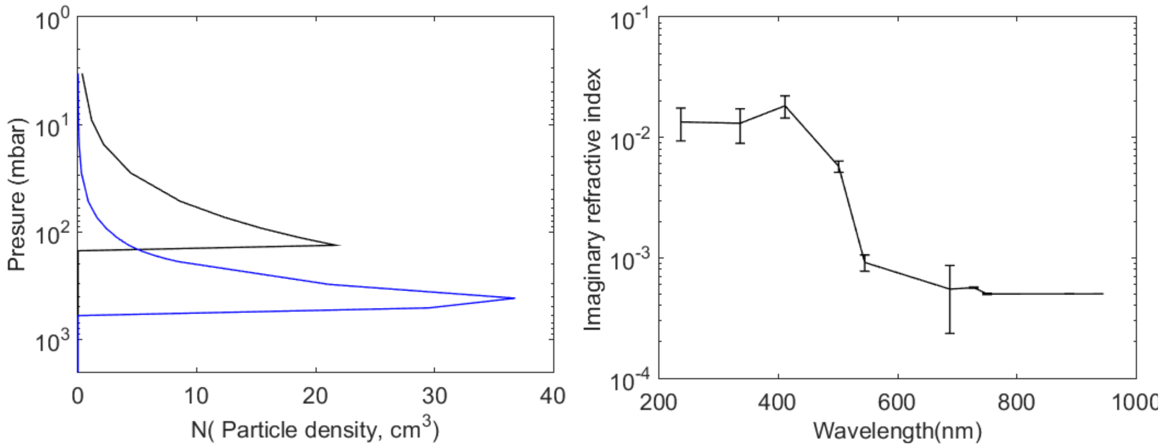


**Supplementary Figure 5. Radiative transfer model for nadir view.** Best-fitting radiative transfer results from the analysis on HST images reflectivity. The images correspond to 29-30 June 2015. Left: retrieved aerosol particle densities as a function of altitude in the atmosphere. The dark line corresponds to the stratospheric haze and the blue line to the tropospheric haze. Note that nadir viewing integrates all layers seen in limb viewing. The two-layer model indicates that both hazes overlap around altitude pressure level of 100 mbar. Right: retrieved refractive imaginary index for the tropospheric particles.

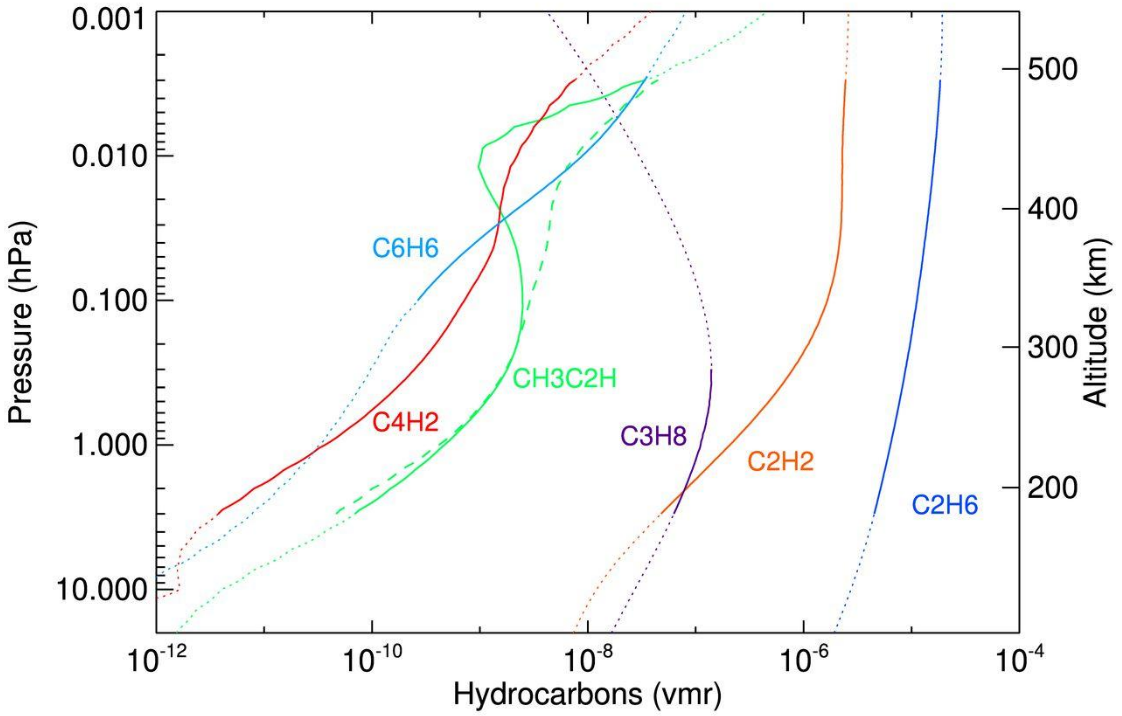


**Supplemetary Figure 6. Vertical hydrocarbons abundance distribution.** Retrieved hydrocarbon vertical distribution (solid lines) on June 16, 2015 from thermal infrared emission spectra at limb obtained with Cassini CIRS. Away from a region of maximum sensitivity, mixing ratio profiles go back to an assumed a priori profile (dotted lines), which are built based on a photochemical model [52]. The dashed green line for CH3C2H represents another potential solution.

# Supplementary Tables

## Supplementary Table 1

*Filters used in the Cassini and HST images*

| **Cassini ISS** (1) | **HST WFPC3** (2) |
|---|---|
| UV1 – 258 nm | F 225W – 225 nm |
| UV2 – 298 nm | F 336W – 336 nm |
| UV3 – 338 nm | F 410M – 410 nm |
| BL1 – 451 nm | F 502N – 502 nm |
| RED – 650 nm | F 547M – 547 nm |
| MT1 – 619 nm (methane abs. band) | F 689M – 689 nm |
| CB1 – 619+ (continuum) | FQ 727N – 727 nm (methane abs. band) |
| MT2 – 727 nm (methane abs. band) | FQ 750N – 750 nm |
| CB2 – 752 nm (continuum) | FQ – 889 nm (methane abs. band) |
| MT3 – 890 nm (methane abs. band) | FQ – 973N (continuum) |
| CB3 – 939 nm (continuum) | |

Notes: (1) Porco, C.C. et al. (2004) [14]; (2) Sanz-Requena, J.F. et al. [15]

## Supplementary Table 2

*List of Cassini ISS images analyzed*

| File Name (COISS 2097) | Date and Time | Filter | Pixel Scale (km px$^{-1}$) | Phase Angle (degrees) | Limb Latitude (degrees) | Limb Longitude (degrees) |
|---|---|---|---|---|---|---|
| N1813144295_1.IMG | 16-6-2015 10:09 | UV1 | 5.01 | 29.1 | 73.5 | 254.0 |
| N1813144327_1.IMG | 16-6-2015 10:09 | UV2 | 2.5 | 29.1 | 73.7 | 253.7 |
| N1813144359_1.IMG | 16-6-2015 10:10 | UV3 | 2.5 | 29.2 | 73.6 | 254.2 |
| N1813144383_1.IMG | 16-6-2015 10:10 | BL1 | 1.25 | 29.3 | 73.7 | 254.3 |
| N1813144454_1.IMG | 16-6-2015 10:12 | RED | 1.25 | 29.4 | 73.6 | 255.0 |
| N1813144538_1.IMG | 16-6-2015 10:13 | MT1 | 1.25 | 29.5 | 73.8 | 255.3 |
| N1813144571_1.IMG | 16-6-2015 10:14 | CB1 | 1.24 | 29.6 | 73.8 | 255.6 |
| N1813144648_1.IMG | 16-6-2015 10:15 | MT2 | 1.24 | 29.7 | 73.5 | 256.7 |
| N1813144721_1.IMG | 16-6-2015 10:16 | CB2 | 1.24 | 29.9 | 73.5 | 257.3 |
| N1813144970_1.IMG | 16-6-2015 10:20 | MT3 | 1.24 | 30.2 | 73.4 | 259.3 |
| N1813145075_1.IMG | 16-6-2015 10:22 | CB3 | 1.23 | 30.5 | 73.3 | 260.2 |
| N1813145136_1.IMG | 16-6-2015 10:23 | CL1 | 1.23 | 30.7 | 73.8 | 259.9 |
